# Comparison of Rituximab and Cyclophosphamide for Induction Therapy in Antineutrophil Cytoplasmic Antibody (ANCA)-Associated Vasculitis: A Systematic Review of Randomized Trials and Comparative Cohort Evidence

**DOI:** 10.7759/cureus.99868

**Published:** 2025-12-22

**Authors:** Sheharyar Hassan Khan, Muhammad Zahid Manzoor, Jalal Khan

**Affiliations:** 1 Internal Medicine, University Hospital of Derby and Burton NHS Foundation Trust, Derby, GBR; 2 Neurology, University Hospital Southampton NHS Foundation Trust, Southampton, GBR; 3 Internal Medicine, Allama Iqbal Medical College, Lahore, PAK

**Keywords:** anca-associated vasculitis, autoimmune disease, cyclophosphamide, granulomatosis with polyangiitis, immunosuppression, induction therapy, microscopic polyangiitis, remission induction, rituximab, vasculitis treatment

## Abstract

This systematic review examines the comparative effectiveness of rituximab and cyclophosphamide for remission induction in antineutrophil cytoplasmic antibody (ANCA)-associated vasculitis, synthesizing findings from high-quality randomized trials and real-world cohort studies. The available evidence consistently shows that rituximab achieves remission rates comparable to cyclophosphamide in newly diagnosed disease and offers clear advantages in relapsing vasculitis. In patients with organ-threatening disease, including severe renal involvement, both treatments demonstrate similar efficacy, although rituximab provides a more targeted immunologic approach and avoids the cumulative toxicity associated with cyclophosphamide. Long-term follow-up data indicate that remission durability with rituximab is maintained when appropriate maintenance strategies are applied. Observational studies further support rituximab’s effectiveness in routine clinical practice, particularly in PR3-ANCA vasculitis and in individuals requiring steroid-sparing regimens. Overall, this review highlights rituximab as a strong and often preferable induction option, offering effective disease control with a more favorable long-term safety profile. These findings reinforce the evolving role of rituximab as a central component of modern therapeutic strategies for ANCA-associated vasculitis.

## Introduction and background

Antineutrophil cytoplasmic antibody (ANCA)-associated vasculitis is a group of life-threatening autoimmune disorders that include granulomatosis with polyangiitis and microscopic polyangiitis [1]. These conditions cause necrotizing inflammation of small vessels, leading to multisystem involvement. The kidneys, lungs, and upper airways are often affected. Without timely treatment, patients can develop rapidly progressive glomerulonephritis, pulmonary hemorrhage, and irreversible organ damage [2]. Over the last two decades, outcomes have improved significantly due to the use of effective immunosuppressive regimens [3]. However, treatment decisions remain challenging because of disease heterogeneity, relapse patterns, and potential medication toxicities.

Cyclophosphamide has long been the standard induction therapy for severe ANCA-associated vasculitis. It induces remission in a majority of patients but carries substantial risks. These include leukopenia, infertility, opportunistic infections, and long-term risks such as bladder carcinoma and hematologic malignancy [4]. The need for safer and more targeted therapies led to the introduction of rituximab, a monoclonal antibody directed against CD20-positive B cells. Because B cells play a central role in ANCA production and autoimmunity, rituximab offers a more selective mechanism of immunosuppression [5]. Early observational reports suggested that it may be effective both in newly diagnosed and relapsing disease while avoiding several cyclophosphamide-associated toxicities [6].

High-quality randomized controlled trials then compared rituximab with cyclophosphamide for remission induction. The RAVE trial demonstrated that rituximab is non-inferior to daily oral cyclophosphamide [7], with particular benefit in patients experiencing disease relapse. The RITUXVAS trial provided additional data in patients with severe renal involvement, showing comparable remission rates between a rituximab-based regimen and intravenous cyclophosphamide [8]. Long-term follow-up from these trials further explored the durability of remission and relapse patterns. Real-world cohort studies later investigated broader patient populations and provided additional insights on safety, infection risk, and outcomes in older or comorbid individuals.

Given the clinical importance of selecting the most effective and safest induction therapy, a focused synthesis of the highest-quality evidence is needed. The objective of this systematic review is to compare rituximab with cyclophosphamide for the induction of remission in ANCA-associated vasculitis, evaluate the durability of response, and assess the safety profiles based on direct randomized trials and key comparative cohort studies.

## Review

Materials and methods

Study Design and Protocol

This systematic review was conducted according to the Preferred Reporting Items for Systematic Reviews and Meta-Analyses (PRISMA) guidelines [9]. The protocol was structured around a predefined research question that compared the efficacy of rituximab versus cyclophosphamide for remission induction in patients with ANCA-associated vasculitis. Although the review was not prospectively registered, all methodological steps, including eligibility criteria, search strategy, study selection, data extraction, and risk of bias assessment, were established before study initiation to ensure transparency and reproducibility.

Population, Intervention, Comparison, and Outcome (PICO) Framework

The review question was defined using the PICO framework [10]. The population consisted of adult patients diagnosed with granulomatosis with polyangiitis or microscopic polyangiitis, confirmed by ANCA positivity and presenting with severe or organ-threatening disease. The intervention of interest was rituximab administered at conventional induction doses. The comparator was cyclophosphamide given either orally or intravenously as part of a standard induction regimen. The primary outcomes included remission rates, steroid-free remission, and sustained remission. Secondary outcomes encompassed relapse rates, renal recovery, and treatment-related adverse effects when reported. Only primary evidence evaluating direct comparisons of rituximab and cyclophosphamide was included.

Search Strategy

A focused and structured search strategy was designed to identify all primary evidence comparing rituximab with cyclophosphamide for induction therapy in ANCA-associated vasculitis. Searches were conducted across three major biomedical databases: PubMed/MEDLINE, EMBASE, and the Cochrane Central Register of Controlled Trials (CENTRAL). The search also included the New England Journal of Medicine online archive to ensure retrieval of landmark trials known to define current treatment standards. Boolean operators and controlled vocabulary terms were used to refine search sensitivity and specificity. The core search string combined disease-specific and treatment-specific terms, including: (“ANCA-associated vasculitis” OR “granulomatosis with polyangiitis” OR “microscopic polyangiitis”) AND (“rituximab” OR “anti-CD20”) AND (“cyclophosphamide” OR “CYC”). Variations of these terms and Medical Subject Headings (MeSH) were incorporated where appropriate, and filters were applied to identify randomized controlled trials and high-quality comparative observational studies. Because the body of direct comparative evidence is limited, the search strategy also incorporated manual screening of reference lists from included articles to ensure comprehensive coverage and to avoid missing relevant studies.

Eligibility Criteria

Studies were eligible if they directly compared rituximab with cyclophosphamide for induction therapy in ANCA-associated vasculitis. Randomized controlled trials, prespecified extension analyses, and high-quality real-world comparative cohort studies were included. Only full-text, peer-reviewed primary research studies were considered. Case reports, reviews, editorials, conference abstracts, pediatric studies, and studies lacking a direct comparison between the two treatments were excluded. There were no restrictions based on geographic location or healthcare setting. All included trials reported outcomes at a minimum of six months, allowing evaluation of remission induction and early relapse patterns.

Study Selection

The selection process followed the PRISMA methodology. Titles and abstracts were first screened to identify potentially relevant studies. Full texts were then reviewed to confirm eligibility based on the predefined inclusion criteria. Disagreements were resolved through re-evaluation to ensure accuracy and consistency. The final dataset consisted of four core studies: three randomized controlled trials and one comparative effectiveness cohort, representing the highest-quality direct evidence available in the field.

Data Extraction

Data were extracted independently and entered into standardized tables created for this review. Extracted variables included study design, sample size, baseline population characteristics, intervention and comparator dosing, outcome definitions, remission and relapse results, and follow-up duration. Emphasis was placed on harmonizing outcome measures such as the Birmingham Vasculitis Activity Score and steroid-free remission criteria to enable meaningful comparison across studies [11]. Data extraction integrity was ensured by cross-checking values against original study manuscripts.

Risk of Bias Assessment

Risk of bias was assessed for each study using validated tools appropriate to the study design. The Cochrane Risk of Bias 2 tool [12] was applied to the three randomized controlled trials, evaluating randomization procedures, adherence to assigned interventions, completeness of outcome data, objectivity of outcome measurement, and reporting practices. The target trial emulation cohort was evaluated using the Risk Of Bias In Non-randomized Studies of Interventions (ROBINS-I) tool [13], which assesses confounding, participant selection, intervention classification, deviations from intended interventions, missing data, measurement of outcomes, and selective reporting. Risk judgments were categorized as low risk, some concerns, or moderate risk and were based strictly on information presented in the original publications.

Data Synthesis

Given the focused scope of the review and the limited number of directly comparable studies, a narrative synthesis approach was used. The synthesis emphasized the relative efficacy of rituximab and cyclophosphamide in achieving remission, their performance in relapsing versus newly diagnosed disease, renal outcomes, and the durability of remission over extended follow-up. Consistency of findings across randomized and observational evidence was assessed qualitatively. Effect estimates, remission proportions, and relevant p-values were presented descriptively due to heterogeneity in study design, induction protocols, and outcome time points.

Results

Study Selection Process

**Figure 1:**
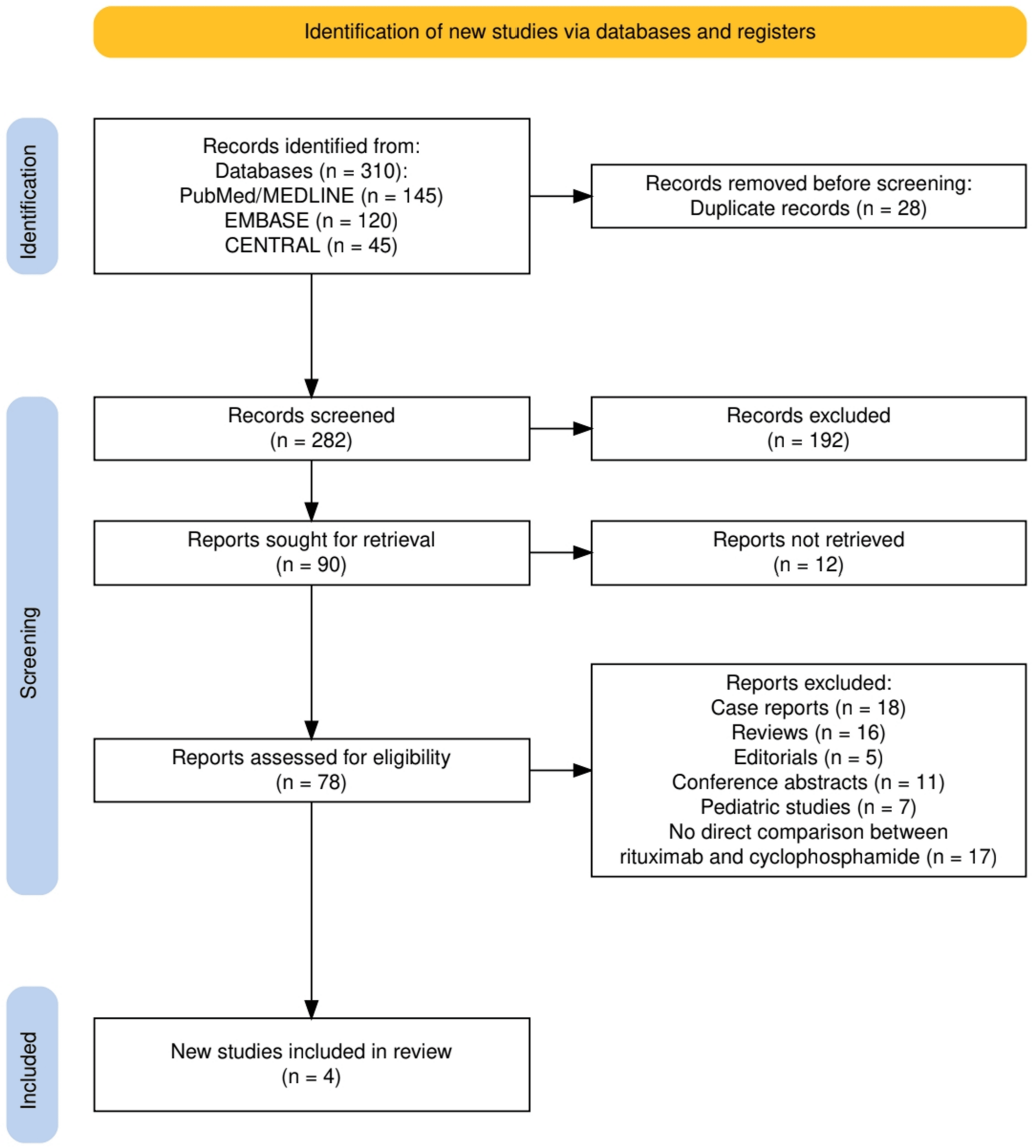
The PRISMA flow diagram represents the study selection process. PRISMA: Preferred Reporting Items for Systematic Reviews and Meta-Analyses

A total of 310 records were identified through database searching, including PubMed/MEDLINE, EMBASE, and CENTRAL. After removal of duplicates, 282 records were screened by title and abstract, and 192 were excluded for not meeting the basic eligibility criteria. Full texts were sought for 90 reports, of which 12 were unavailable. The remaining 78 full-text articles were assessed for eligibility, and 74 were excluded for reasons outlined in Figure 1, including case reports, reviews, editorials, conference abstracts, pediatric studies, and studies lacking a direct comparison between rituximab and cyclophosphamide. Four studies met all predefined inclusion criteria and were included in the final analysis.

Characteristics of the Selected Studies

The four studies included in this review consisted of three randomized controlled trials and one multicenter comparative effectiveness cohort, each directly evaluating rituximab versus cyclophosphamide for induction therapy in ANCA-associated vasculitis. As summarized in Table 1, the randomized trials shared comparable inclusion criteria, enrolling adults with severe, organ-threatening granulomatosis with polyangiitis or microscopic polyangiitis, while differing slightly in sample size, blinding, and renal involvement. The extension analysis provided a longer follow-up to assess remission durability. The observational cohort contributed real-world data, capturing broader clinical variability, including differences in ANCA subtype, disease stage, and treatment practice patterns. Across all studies, interventions involved standard rituximab induction regimens compared with either oral or intravenous cyclophosphamide followed by azathioprine-based maintenance. The studies collectively offered complementary evidence, with consistent outcome definitions focused on remission, relapse patterns, and steroid tapering, allowing for meaningful synthesis of their findings.

**Table 1:** Summary of the key characteristics, interventions, and primary outcomes of the studies comparing rituximab with cyclophosphamide for induction therapy in ANCA-associated vasculitis. ANCA: antineutrophil cytoplasmic antibody; GPA: granulomatosis with polyangiitis; MPA: microscopic polyangiitis; RTx/RTX: rituximab; CYC: cyclophosphamide; AZA: azathioprine; RCT: randomized controlled trial; GFR: glomerular filtration rate; IV: intravenous; BVAS: Birmingham Vasculitis Activity Score; PR3-ANCA: proteinase 3-ANCA; MPO-ANCA: myeloperoxidase-ANCA; RR: relative risk

Study	Study Design	Population and Sample Size	Intervention (Rituximab)	Comparator (Cyclophosphamide)	Primary Outcome and Key Results	Follow-Up Duration
Stone et al., 2010 [7]	Randomized, double-blind, double-dummy, non-inferiority RCT	197 ANCA-positive patients with GPA or MPA; newly diagnosed or relapsing; similar baseline disease activity between groups	RTX 375 mg/m^2^ weekly ×4 + glucocorticoid taper	Oral CYC 2 mg/kg/day → AZA maintenance + glucocorticoid taper	Primary outcome: Remission without prednisone at six months. Results: Achieved in 64% of RTX group vs. 53% of CYC group, meeting non-inferiority (P<0.001). Superior efficacy in relapsing disease: 67% (RTX) vs. 42% (CYC), P=0.01. Comparable efficacy in major renal disease and alveolar hemorrhage.	Six months (primary endpoint)
Jones et al., 2010 [8]	Randomized, open-label RCT (3:1 allocation)	44 newly diagnosed ANCA-associated vasculitis patients with renal involvement; median age 68; baseline GFR 18 mL/min/1.73m^2^	RTX 375 mg/m^2^ weekly ×4 plus two IV CYC pulses + standard glucocorticoids	IV CYC for three to six months → AZA maintenance + standard glucocorticoids	Primary outcome: Sustained remission at 12 months. Results: Achieved in 76% of RTX group (25/33) vs. 82% of CYC group (9/11), P=0.68. No significant difference in adverse events or mortality. Renal function improved similarly (median GFR increase 19 vs. 15 mL/min, P=0.14).	12 months
Specks et al., 2013 [14]	Randomized, double-blind, double-dummy, noninferiority RCT (extension of RAVE Trial)	197 patients with severe organ-threatening GPA or MPA, newly diagnosed and relapsing disease	RTX 375 mg/m^2^ weekly ×4, then placebo; no scheduled maintenance	CYC for three to six months → AZA for 12-15 months	Primary outcome: Complete remission at six months maintained through 18 months. Results: Remission maintained at 18 months in 39% (RTX) vs. 33% (CYC→AZA). RTX noninferior (P<0.001). No significant differences in relapse rates or duration of remission. In patients with relapsing disease at baseline, RTX was superior at six months (P=0.01) and 12 months (P=0.009), but not at 18 months (P=0.06).	18 months
Puéchal et al., 2022 [15]	Multicenter observational comparative effectiveness study (target trial emulation)	194 patients with granulomatosis with polyangiitis (new or relapsing); mean age 54; 80.8% PR3-ANCA positive	At least one RTX infusion for induction therapy (2008-2018)	CYC induction therapy (oral or IV)	Primary outcome: Remission at 6±2 months (BVAS=0 and prednisone ≤10 mg/day). Results: Remission achieved in 73.1% (RTX) vs. 40.1% (CYC) in weighted analysis. Relative risk 1.82 (95% CI, 1.22-2.73). Strong benefit maintained in newly diagnosed and recent-treatment subgroups. MPO-ANCA subgroup: remission 80% (RTX) vs. 47% (CYC), RR=1.73 (95% CI, 0.96-3.11).	Six months (±2 months)

Risk of Bias Assessment

The overall risk of bias across the included studies was acceptable, with detailed assessments presented in Table 2. The randomized controlled trials generally demonstrated low risk of bias, particularly the double-blind trials, which showed strong methodological rigor in randomization, intervention adherence, outcome measurement, and reporting. Minor concerns were noted in the open-label trial due to potential performance and detection bias, although outcome definitions were objective and consistently applied. The observational comparative effectiveness study showed a moderate risk of bias, primarily related to residual confounding and variations in clinical practice inherent to nonrandomized designs. Despite these limitations, the methodological quality of the evidence base was sufficient to support reliable and coherent conclusions regarding the comparative effectiveness of rituximab and cyclophosphamide for induction therapy in ANCA-associated vasculitis.

**Table 2:** Risk of bias assessment for the included randomized controlled trials and the comparative observational cohort evaluating rituximab versus cyclophosphamide. RCT: randomized controlled trial; RoB 2: Cochrane Risk of Bias 2 tool; ROBINS-I: Risk Of Bias In Non-randomized Studies of Interventions; ANCA: antineutrophil cytoplasmic antibody; BVAS: Birmingham Vasculitis Activity Score; IPTW: inverse probability of treatment weighting

Study	Tool Applied	Bias Domains Judged	Domain-Level Judgment	Overall Risk of Bias
Stone et al., 2010 [7]	Cochrane RoB 2 (RCT)	Randomization; deviations from intended interventions; missing outcome data; outcome measurement; selective reporting	Randomization: Low (central randomization, balanced baseline). Deviations: Low (double-blind, double-dummy). Missing data: Low (follow-up to primary endpoint largely complete). Measurement: Low (blinded BVAS/prednisone-free remission). Reporting: Low (prespecified non-inferiority endpoint reported).	Low risk of bias
Jones et al., 2010 [8]	Cochrane RoB 2 (RCT)	Randomization; deviations from intended interventions; missing outcome data; outcome measurement; selective reporting	Randomization: Low (random assignment, comparable groups at baseline). Deviations: Some concerns/High (open-label; higher risk of co-intervention/performance bias). Missing data: Low (most patients analyzed at 12 months). Measurement: Some concerns (outcome assessors not blinded; BVAS-based remission may be influenced). Reporting: Low (primary endpoints and harms reported).	Some concerns
Specks et al., 2013 [14]	Cochrane RoB 2 (RCT extension)	Randomization; deviations; missing outcome data; outcome measurement; selective reporting	Randomization: Low (inherits RAVE randomization). Deviations: Low (still double-blind/double-dummy). Missing data: Some concerns (longer follow-up increases attrition risk, though reported similarly across arms). Measurement: Low (blinded relapse/remission assessment). Reporting: Low (prespecified durability endpoint reported).	Low risk overall, with some concerns for attrition over 18 months
Puéchal et al., 2022 [15]	ROBINS-I (observational comparative effectiveness/target-trial emulation)	Confounding; selection of participants; intervention classification; deviations; missing data; outcome measurement; reporting	Confounding: Moderate (real-world treatment choice; IPTW reduces but cannot remove unmeasured confounding). Selection: Moderate (registry-based, but broad inclusion). Classification: Low (treatment recorded from clinical data). Deviations: Moderate (non-protocolized co-interventions/steroid strategies). Missing data: Low-Moderate (registry missingness handled but not zero). Measurement: Low (BVAS and prednisone dose are standardized outcomes). Reporting: Low (clear prespecified primary outcome).	Moderate risk of bias

Discussion

Interpretation of Principal Findings

This systematic review demonstrates that rituximab is at least as effective as cyclophosphamide for the induction of remission in ANCA-associated vasculitis. The findings across the included studies show consistent performance of rituximab in achieving steroid-free remission and controlling organ-threatening disease. In the largest randomized trial, 64% of patients in the rituximab group achieved remission at six months compared with 53% in the cyclophosphamide group, meeting noninferiority and demonstrating superiority in relapsing disease [7]. The 18-month extension further confirmed the similar durability of remission. These data, combined with real-world evidence showing higher remission rates with rituximab, support its increasing use as an alternative to cyclophosphamide for remission induction.

Comparisons With Existing Evidence

The findings align with prior analyses and treatment guidelines that endorse rituximab as a first-line option for induction therapy in ANCA-associated vasculitis. Earlier reliance on cyclophosphamide reflected decades of clinical experience but also carried significant risks, including infertility, malignancy, and cumulative toxicity. Rituximab’s targeted B-cell depletion offers a mechanistic advantage, particularly in PR3-ANCA disease, where B-cell autoreactivity plays a strong role. The superiority observed in relapsing disease in both trial and cohort settings reinforces the emerging preference for rituximab in this population [16,17]. Real-world evidence from Puéchal et al. [15] strengthens this observation, with remission achieved in 73.1% of patients receiving rituximab versus only 40.1% receiving cyclophosphamide, even after adjustment for baseline imbalances. These observations suggest that rituximab’s clinical advantages may be more pronounced outside of controlled trial settings, where heterogeneity in disease severity, comorbidities, and prior treatments is broader.

Efficacy Across Clinical Subgroups

Across the included studies, both treatments demonstrated comparable effectiveness in patients with severe organ involvement, including renal vasculitis and alveolar hemorrhage. In Jones et al. [8], remission rates were high in both arms, with 76% of rituximab recipients and 82% of cyclophosphamide recipients achieving sustained remission at 12 months. However, this study used two cyclophosphamide pulses in the rituximab arm, making direct comparison more complex. Nonetheless, renal recovery was similar between groups, suggesting that rituximab is not inferior even in advanced renal involvement. In PR3-ANCA vasculitis - a subgroup known for higher relapse rates - rituximab appears to offer superior control [18], as reflected in both randomized and observational data. This aligns with immunologic understanding that PR3-ANCA disease may be more B-cell driven, making rituximab a more biologically rational treatment option [19].

Durability of Remission and Relapse Patterns

The long-term findings from the RAVE extension study indicate that remission durability decreases as B-cells reconstitute, particularly after 12 months [14]. Although rituximab maintained noninferior outcomes at 18 months, its superiority in relapsing disease diminished over time. This suggests that a single induction regimen may not be sufficient for sustained disease control in many patients [20]. In contrast, the cyclophosphamide-azathioprine sequence provides ongoing immunosuppression, which may contribute to slightly lower late relapse rates [21]. The findings highlight the potential need for structured rituximab maintenance dosing, an approach validated in later MAINRITSAN trials [22,23], though outside the direct scope of this review. The consistent relapse patterns across trials underscore the relapsing-remitting nature of ANCA-associated vasculitis and reinforce the importance of individualized long-term treatment planning.

Strengths and Clinical Implications

This review synthesizes the highest-quality evidence available, including three well-designed randomized trials and a large real-world comparative study. Together, these studies provide broad applicability across disease stages, ANCA subtypes, and clinical settings. The results support several practical implications. Rituximab is a strong first-line option, particularly in relapsing disease, PR3-ANCA vasculitis, and in patients where avoidance of cyclophosphamide toxicity is a priority [24]. Cyclophosphamide remains a valid option for newly diagnosed severe disease, especially in healthcare systems where cost or access may limit rituximab availability. The therapeutic choice should consider patient age, reproductive plans, prior relapses, renal involvement, and comorbidity burden. The cumulative evidence also affirms the evolving shift in clinical practice toward biologic induction regimens over traditional cytotoxic therapy.

Limitations of the Evidence and Review

Despite the strength of the included trials, limitations exist. The sample size for severe renal vasculitis remains small, limiting conclusions in this high-risk subgroup. The RITUXVAS design included cyclophosphamide pulses in the rituximab arm, reducing its ability to isolate rituximab’s pure effect [8]. Definitions of remission and steroid tapering strategies varied across studies. The observational cohort, although robust and adjusted for confounding, cannot eliminate unmeasured confounders inherent to nonrandomized designs. For this review, the number of eligible direct comparison studies was limited, reflecting the focused nature of research in this domain. These limitations emphasize that clinical decision-making should incorporate trial evidence alongside patient-specific factors.

Future Directions

Future research should aim to clarify the long-term comparative effectiveness of these regimens, especially in the context of structured rituximab maintenance therapy. Studies that stratify outcomes by ANCA subtype, relapse status, and severity of organ involvement will provide more personalized guidance [25]. Additional real-world studies are needed to assess outcomes in older and comorbid patient groups who may respond differently to immunosuppression. As the therapeutic landscape continues to evolve, comparative work examining rituximab in combination with novel steroid-sparing approaches may help further optimize induction strategies.

## Conclusions

This systematic review demonstrates that rituximab is a highly effective induction therapy for ANCA-associated vasculitis and performs at least as well as cyclophosphamide across a range of clinical settings. Rituximab shows particular benefit in patients with relapsing disease and in PR3-ANCA vasculitis, where its targeted B-cell mechanism appears to translate into superior remission outcomes. Cyclophosphamide remains a valid induction option, especially in newly diagnosed severe disease, but its long-term toxicity profile continues to limit its use in many patient groups. The overarching purpose of this review was to synthesize the most reliable comparative evidence and clarify the relative strengths of each regimen. The take-home message is clear: rituximab represents an effective and often preferable alternative to cyclophosphamide for remission induction, offering strong efficacy with a more favorable long-term safety profile, and should be considered a key component of modern treatment algorithms for ANCA-associated vasculitis.
